# miRNA Expression in Fibroblastic Foci within Idiopathic Pulmonary Fibrosis Lungs Reveals Novel Disease-Relevant Pathways

**DOI:** 10.1016/j.ajpath.2022.12.015

**Published:** 2023-01-20

**Authors:** Laura Sabater, Jean B. Gossart, Inmaculada Hernandez, Daniel Rico, Andy Blanchard, Lee A. Borthwick, Andrew J. Fisher, Joaquim Majo, Kasim Jiwa, Amy Collins, Giuseppe Abbate, Fiona Oakley, Derek A. Mann, Jelena Mann

**Affiliations:** ∗ Newcastle Fibrosis Research Group, Biosciences Institute, Newcastle University, Newcastle upon Tyne, United Kingdom; † Computational Epigenomics Laboratory, Biosciences Institute, Newcastle University, Newcastle upon Tyne, United Kingdom; ‡ GlaxoSmithKline Medicines Research Centre, Stevenage, United Kingdom; § Institute of Transplantation, Newcastle upon Tyne Hospitals NHS Foundation Trust, Newcastle upon Tyne, United Kingdom; ¶ FibroFind Ltd, FibroFind Laboratories, Medical School, Newcastle University, Newcastle upon Tyne, United Kingdom

## Abstract

miRNAs are 22 nucleotides long and belong to a class of noncoding RNAs that plays an important role in regulating gene expression at a post-transcriptional level. Studies show aberrant levels of miRNAs to be associated with profibrotic processes in idiopathic pulmonary fibrosis (IPF). However, most of these studies used whole IPF tissue or *in vitro* monocultures in which fibrosis was artificially induced. The current study used laser microdissection to collect fibroblastic foci (FF), the key pathologic lesion in IPF, isolated miRNAs, and compared their expression levels with those found in whole IPF lung tissue and/or *in vitro* cultured fibroblast from IPF or normal lungs. Sequencing libraries were generated, and data generated were bioinformatically analyzed. A total of 18 miRNAs were significantly overexpressed in FF tissue when compared with whole IPF tissue. Of those, 15 were unique to FF. Comparison of FF with cultured IPF fibroblasts also revealed differences in miRNA composition that impacted several signaling pathways. The miRNA composition of FF is both overlapping and distinct from that of whole IPF tissue or cultured IPF fibroblasts and highlights the importance of characterizing FF biology as a phenotypically and functionally discrete tissue microenvironment.

Idiopathic pulmonary fibrosis (IPF) is a chronic, progressive fibrotic lung disease characterized by a restrictive ventilatory defect and impaired gas transfer due to deposition of fibrotic tissue in the lung interstitium. The incidence of IPF has been reported as ranging from 2.8 to 18 cases per 100,000.^1^ With 6000 new patients/year presenting and a disease prevalence of approximately 32,000 in the United Kingdom (British Lung Foundation UK IPF Statistics, *https://www.blf.org.uk/support-for-you/idiopathic-pulmonary-fibrosis-ipf/statistics*, last accessed February 2, 2022), it appears to be increasing steadily. IPF has a poor prognosis, with a median survival of 2 to 4 years from diagnosis, making post-diagnosis survival worse than many cancers.^2^

The etiology of IPF remains unclear, but growing evidence points towards complex interactions between genetic risk factors and environmental insults on a background of age-associated predisposition as the key contributors.^3^, ^4^, ^5^ Treatment options for IPF are limited and are predominately palliative. Two distinct pharmaceutical agents, pirfenidone and nintedanib, are licensed as novel IPF treatments. However, at best, these agents decrease the rate of decline in patient lung function and reduce the risk of acute deteriorations of lung function rather than halt or reverse the fibrogenic process.^6^^,^^7^ Lung transplantation is the only option that offers hope for long-term survival, but it is only available to highly selected individuals.^8^

The pathology of IPF is characterized by disruption of normal lung architecture due to deposition of excessive collagen and extracellular matrix in the alveolar walls, and development of aggregates of proliferating fibroblasts and myofibroblasts, which are recognized as fibroblastic foci (FF) on histologic evaluation of diseased tissue.^9^^,^^10^ The foci represent discrete sites of lung injury and repair and are of pivotal importance to the progression of IPF. An improved understanding of the mechanisms that lead the fibroblast/myofibroblast population within the FF in the lung to proliferate and produce excessive extracellular matrix is critical to identifying potential pathways to target new therapies that might halt or even reverse the disease process.

miRNAs are short noncoding RNAs that regulate gene expression in a post-transcriptional manner through binding to the 3′-untranslated region of their target mRNAs. This interferes with protein production by destabilizing the mRNA and causing translational fine-tuning. As a result, miRNA expression levels can influence several cellular processes, including differentiation, proliferation, activation, and apoptosis.^11^ The altered levels of miRNAs have been investigated in several fibrotic tissues in both animal models and human disease and have been associated with fibrosis progression.^12^ However, many of these studies have used whole organ tissues, comparing the diseased organ with the normal healthy control. Alternatively, studies were performed on single-cell types, predominantly epithelium or fibroblasts, cultured from diseased or normal tissue or from animal organs in which fibrosis has been artificially induced.^13^ All of these approaches carry inherent problems associated with the heterogeneity of cell types and their ratios in the diseased versus healthy organ context or issues emanating from isolated cells cultured on stiff tissue culture plastic.

This study focused on improving our understanding of the role of miRNAs in the pathophysiology of IPF by investigating the expression patterns of miRNAs within FF themselves, the hallmark lesions of IPF that are rich in fibroblasts and myofibroblasts, which are cell types known to drive fibrogenesis. To do this, multiple foci from IPF lung tissue samples were collected using laser capture microdissection (LCM), and the miRNA profile was quantified by next-generation sequencing. The levels of miRNAs in FF were compared with those found in total IPF tissue or in fibroblast cultures isolated from matched IPF or normal control lung tissue to investigate whether the process of culturing cells on plastic altered the miRNA levels. Combining these approaches, the study uncovered novel pathways operating within FF that have not previously been described.

## Materials and Methods

### IPF Lung Tissue Preparation for Laser Microdissection

Formalin-fixed, paraffin-embedded blocks of IPF lung tissue from nine patients were sectioned and stained with Mayer’s hematoxylin. The slides were used for LCM isolation of FF on the same day. FF were detected and selected by a pathologist (J.Maj.. and K.J.) and later confirmed and cut out using LCM by pathology-trained technician.

### Human Subjects

Use of human tissue was approved by Newcastle and North Tyneside Local Research Ethics number 11/NE/0291. All samples were collected and used subject to patient's written consent. The study was conducted in accordance with the Declaration of Helsinki (as revised in 2013). IPF tissue was obtained from patients undergoing lung transplantation at the Institute of Transplantation, Newcastle Upon Tyne Hospitals NHS Foundation Trust.

### Laser Microdissection of IPF Lung Tissue

The FF were cut from the IPF tissue sections using Zeiss Laser Capture Microdissection Microscope (Zeiss, Carl Zeiss Microscopy GmbH, Jena, Germany). The area of interest was cut out using an automated laser pressure catapulting method. Microdissected areas were collected in an AdhesiveCap (Zeiss, Carl Zeiss Microscopy GmbH), and RNA was isolated using RNeasy FFPE kit (Qiagen, Hilden, Germany), as per manufacturer's instructions. The surface area of 9,000,000 ± 1,000,000 μm^2^ was found to generate 50 to 200 ng of RNA, which comprised at least 70 to 90 pooled individual foci. The foci were pooled from the same lung only, and never between patients. Total RNAs were extracted using FFPE RNeasy extraction kit (Qiagen), according to the manufacturer's protocol.

### Small RNA Library Preparation and Sequencing

For each patient, NEBNext small RNA libraries for next-generation sequencing (New England Bio Labs Inc., Ipswich, MA) were prepared from total RNA. QIAquick PCR purification kit (Qiagen) and a 6% polyacrylamide gel were used to perform the library quality control and size selection of 21-nucleotide RNA fragments. Qubit dsDNA HS Assay kit and a Qubit 2.0 fluorometer (Life Technologies, Carlsbad, CA) were used to measure the abundance of the libraries, and the size of the fragments contained in the libraries was measured with a DNA high-sensitivity chip and an Agilent 2100 Bioanalyser (Agilent Technologies, Santa Clara, CA). The libraries were sequenced on Illumina (San Diego, CA) MiSeq, according to the manufacturer's protocols at 50-bp read length.

### Isolation and Cell Culture of Fibroblasts from IPF Lung or Control Normal Human Lung and RNA Isolation

Lung fibroblasts were isolated from donor-matched IPF lung tissue that was used for LCM and grown on plastic. Five fibroblast lines were further grown by the outgrowth method from normal human lungs and were used as controls. The cells were grown to 90% confluence, then serum starved for 24 hours in media containing 0.4% fetal calf serum. After the 24-hour period, the cells were incubated for 48 hours with complete media only or media supplemented with 3 ng/mL transforming growth factor (TGF)-β1. The cells were harvested at 48 hours, and total RNA was extracted using the RNeasy mini kit (Qiagen).

### Small RNA Data Processing

FASTQ files obtained from a run on MiSeq were trimmed, size selected, and mapped with ChimiRa release 1.0^14^ (*http://wwwdev.ebi.ac.uk/enright-dev/chimiRa/index.php*, last accessed March 24, 2020). ChimiRa was used to trim the sequences from sequencing adapters, using as adapter sequence AGATCGGAAGAGC, then map them against human miRNA hairpin sequences from miRBase, and extract count-based miRNA expression data.^14^ Counts were analyzed using R statistics (*https://www.r-project.org*) and normalized using DESeq2 package for R version 3.0.1^15^; heat maps and volcano plots (fold change > 1 and *P* = 0.01) were plotted for all samples and conditions using gplots package for R version 3.0.1 (*https://cran.r-project.org/web/packages/gplots/gplots.pdf*).

### Availability of Data and Material

Raw and processed miRNA sequencing data can be found at Gene Expression Omnibus using the accession number GSE220107 (*https://www.ncbi.nlm.nih.gov/geo*).

## Results

**Figure 1:**
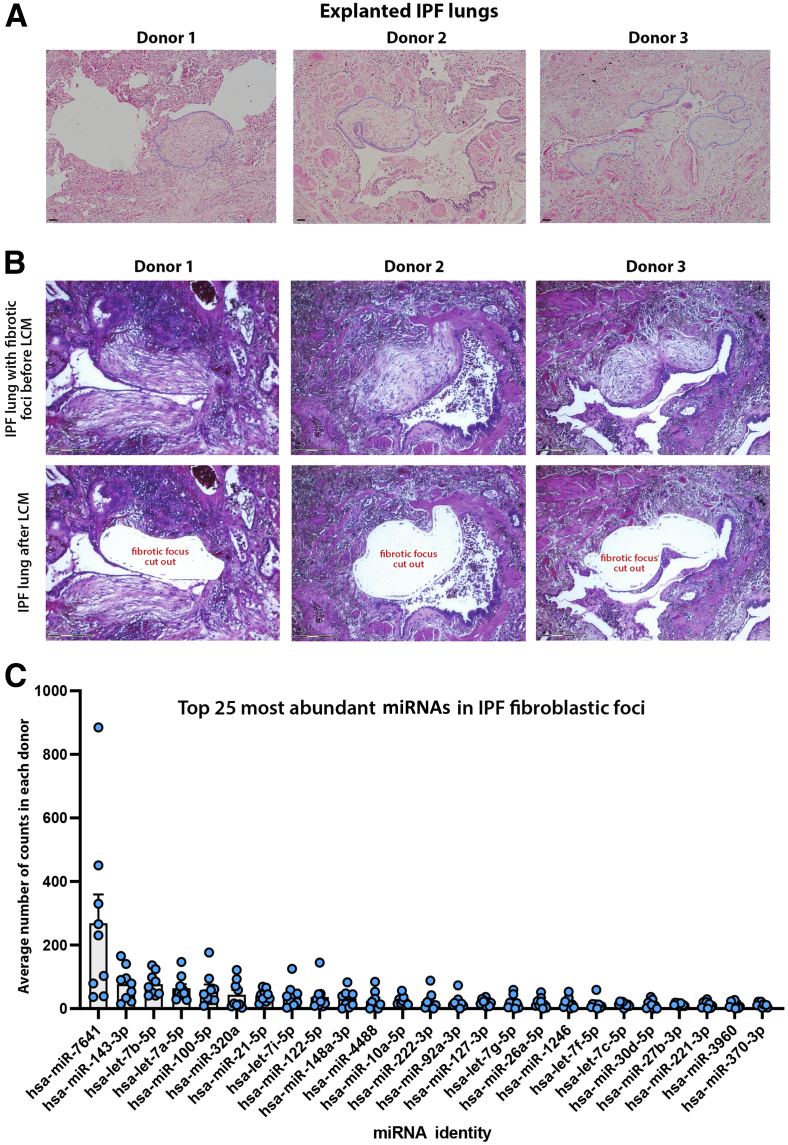
Fibroblastic foci identification from histology sections of idiopathic pulmonary fibrosis (IPF) explanted lungs. **A:** Representative images of hematoxylin and eosin–stained IPF explanted lungs showing fibroblastic foci outlined in **light blue dotted line**. **B:** Representative images of the identification and laser cut microdissected fibroblastic foci in hematoxylin IPF lung sections. **Top panels:** Fibroblastic foci identification in hematoxylin IPF lung sections before laser capture microdissection (LCM). **Bottom panels:** Matched IPF lung sections after LCM of the fibroblastic foci. **C:** Top 25 most expressed miRNAs in IPF fibroblastic foci. Scale bars: 100 μm (**A**); 150 μm (**B**).

**Figure 2:**
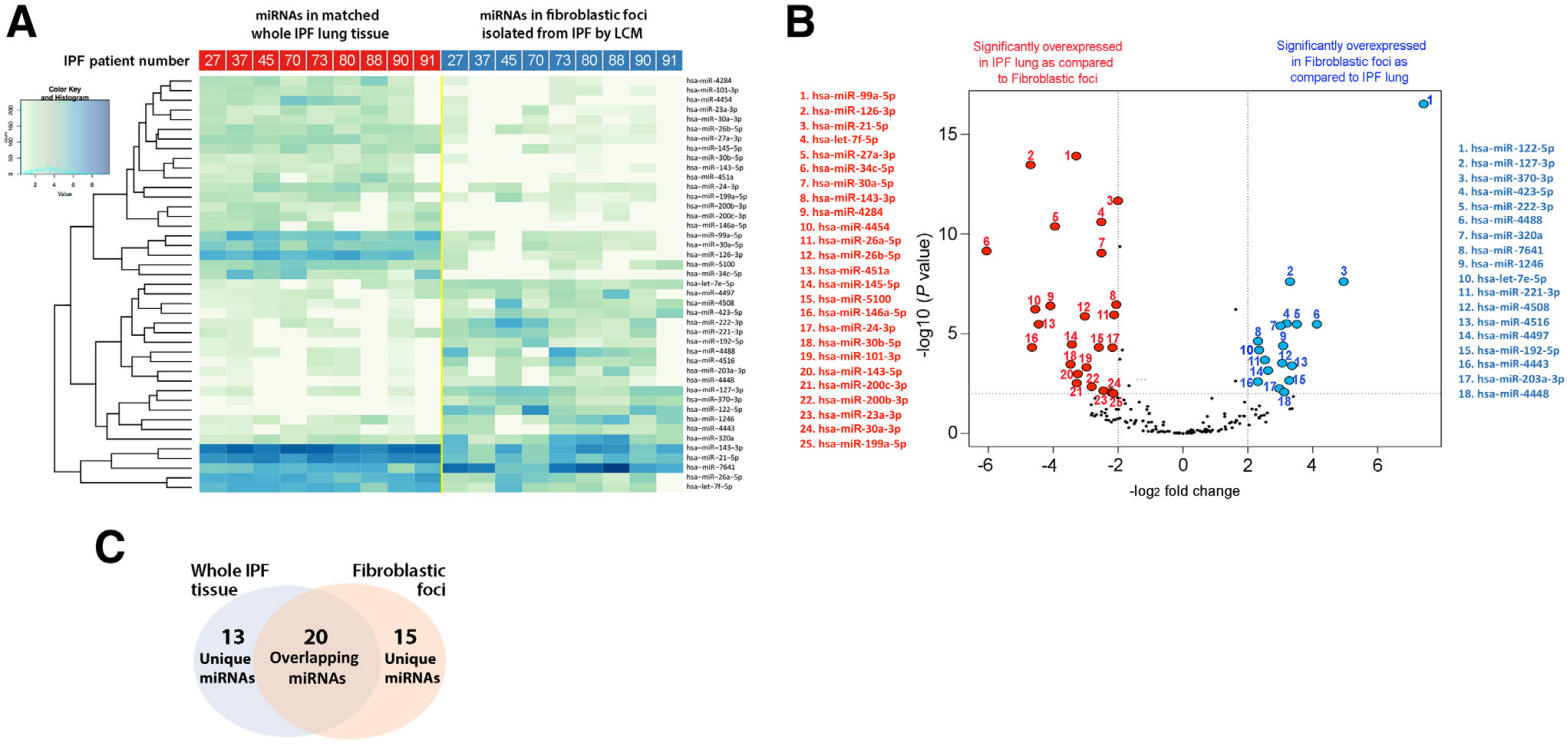
Differential expression of miRNAs in human idiopathic pulmonary fibrosis (IPF) lungs and fibroblastic foci (FF) isolated from the same lungs (heat map). **A:** Heat map of the 43 significant differently expressed miRNAs in the comparison between fibroblastic foci isolated from IPF lungs (**blue; right side**) and matched whole IPF lung tissue (**red; left side**); *P* < 0.01 and fold change >2. Color scale from pale green to dark blue being lower to higher expression, respectively. **B:** Volcano plot of the significantly differently expressed miRNAs. Significantly overexpressed miRNAs in fibroblastic foci are indicated in **blue**. Significantly overexpressed miRNAs in whole IPF lung are indicated in **red**. *P* < 0.01 and fold change >2. **C:** Venn diagram of the miRNAs with >10 counts in at least three samples per group. **Pale orange** indicates unique miRNAs in fibroblastic foci, **pale blue** indicates unique miRNAs in whole IPF tissue, and **overlapping area** indicates miRNAs found in common in both FF and whole IPF tissue. LCM, laser capture microdissection.

**Figure 3:**
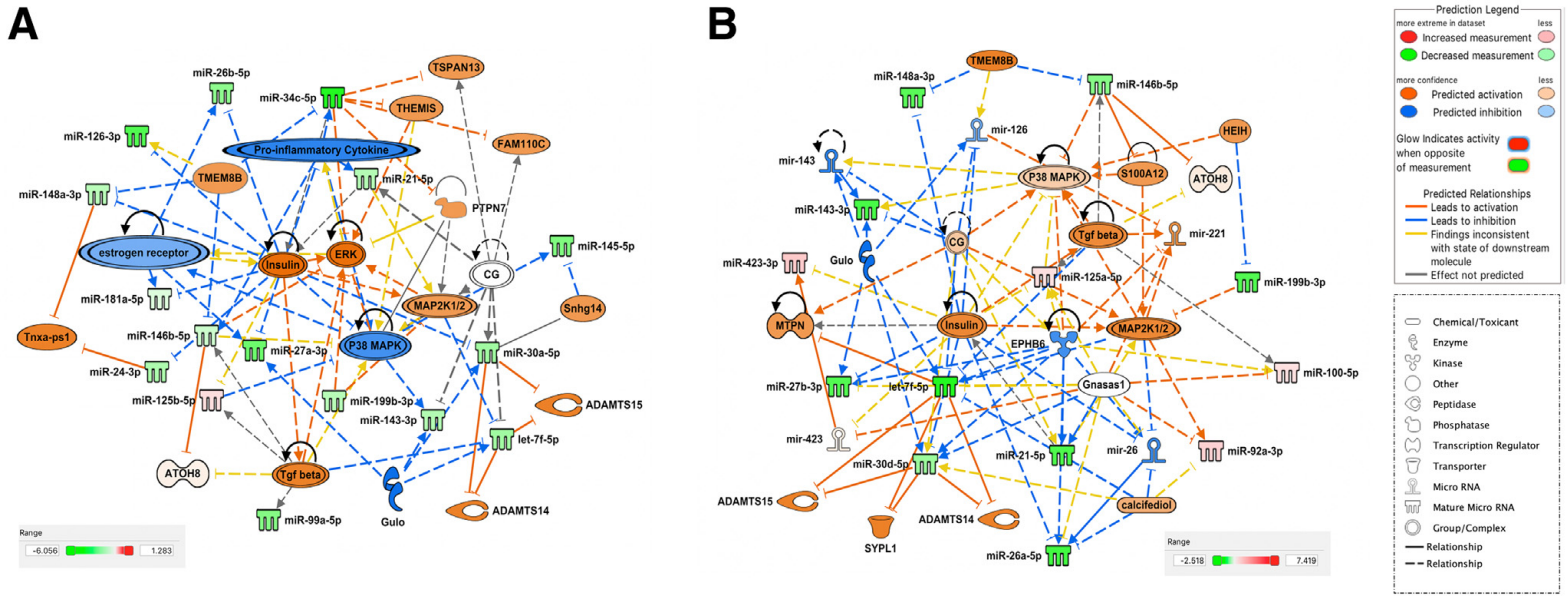
Differential expression of miRNAs in human idiopathic pulmonary fibrosis (IPF) lungs and fibroblastic foci isolated from the same lungs [Ingenuity Pathway Analysis (IPA) plots]. IPA-generated biological network of differentially expressed miRNAs found in whole IPF lung tissue (**A**) and fibroblastic foci (**B**). Underexpressed and overexpressed miRNAs are in **green** and **red**, respectively. Prediction of activation and inhibition is in **orange** and **blue**, respectively. Molecules related to miRNAs for which no prediction of directional change could be made are indicated in **white**. **Lines** represent different relationships between the molecules and miRNAs, as stated in the legend.

To determine the miRNA content of FF, histologic sections of explanted IPF lungs were obtained from nine patients (Table 1). Sections were stained with hematoxylin and eosin, and foci were identified using light microscopy (Figure 1A). Despite some interpatient variability in the numbers of FF present within different explanted IPF lungs, 50 to 200 foci were identified and dissected out from each lung using LCM (Figure 1B). RNA isolated from foci was sequenced, generating on average 3 to 4 million reads for each donor lung. The sequences were mapped onto the human genome, and the number of individual miRNAs sequenced was quantified. The mean of counts for each miRNA shows the most abundant miRNA species expressed in the FF (Figure 1C). RNA isolated from matched whole IPF lung tissue was sequenced next and a direct comparison of miRNA signatures in the LCM isolated FF from the same lung was made, generating a heat map with 43 significantly different miRNAs (Figure 2A and Supplemental Table S1). This direct comparison highlighted 25 miRNAs that were significantly overexpressed in the whole IPF lung tissue compared with the foci (Figure 2B), and 18 miRNAs that are significantly overexpressed in FF compared with the whole IPF lung tissue (Figure 2B), based on a fold change of two or greater and *P* < 0.01. Although there were significant differences in the levels of their expression, 20 miRNAs were present in both samples, with 13 miRNAs unique to IPF lung tissue and 15 miRNAs unique to fibroblastic foci (Figure 2C). To put these miRNAs into a biological context, Ingenuity Pathway Analysis (Qiagen) was performed. Network analysis revealed numerous miRNAs related to IPF and inflammation, as evidenced by their relationship with proinflammatory cytokines, TGF-β, *THEMIS*, and kinases, such as mitogen-activated protein kinase kinase 1 and 2 (MAP2K1/2), extracellular signal-regulated kinase, and p38 mitogen-activated protein kinase. Moreover, miRNAs present in the whole lung were related to regulation of multiple features, such as ADAM metallopeptidase with thrombospondin type 1 motif 14 (ADAMTS14) and ADAMTS15 peptidases, the transcription regulator protein atonal homolog (ATOH8), transmembrane proteins [tetraspanin (TSPAN13) and transmembrane protein 8B (TMEM8B)], insulin, chorionic gonadotropin hormone, estrogen receptor, and other features [protein tyrosine phosphatase non-receptor type 7 (PTPN7), long intergenic non-coding RNA gene FAM110C, long non-coding RNA molecule TNXA-PS1, l-gulono-gamma-lactone oxidase (GULO), and Snhg14] (Figure 3A). However, network analyses based on miRNAs found in LCM isolated fibroblastic foci revealed several molecules not found in the analyses based on whole IPF tissue (Figure 3B). This analysis predicted that pathways such as TGF-β, which are classically associated with tissue fibrosis, were activated, and also identified S100A12 and tetrahydromethanopterin:alpha-l-glutamate ligase (MTPN). Serum levels of S100A12 are negatively associated with lung function in systemic scleroderma^16^ and IPF,^17^ whereas MTPN is reported to convert p65:p50 heterodimers to repressive p50:p50 homodimers, thus differentially regulating NF-κB target genes.^18^ Moreover, other features, such as hepatocellular carcinoma up-regulated EZH2-associated long non-coding RNA (HEIH), kinase epoxide hydrolase B6 (EPHB6), calcifediol, synaptophysin-like protein 1 (SYPL1) transporter, and several miRNAs (miR-126, miR-143, miR-221, miR-26, and miR-423), were also predicted to be associated to miRNAs from LCM isolated FF but were not found in network analysis based on whole lung (Figure 3B).

**Table 1:** Explanted IPF Lungs: Patient Characteristics

Identifier	Sex	Age, years	FEV1, L	FVC, L	TLC, L	TLCO, mmol CO/min/kPa	KCO, mmol CO/min/kPa	Pack years
80	M	54	1.97 (52%)	2.38 (50%)	4.02 (54%)	3.08 (29%)	0.87 (61%)	
27	M	56	2.37 (61%)	2.9 (59%)	4.84 (63%)	2.9 (25%)	0.7 (49%)	Nil
70	M	62	1.43 (51%)	1.79 (51%)	2.73 (45%)	2.12 (26%)	0.85 (63%)	Nil
45	M	44	2.6 (54%)	2.29 (50%)	3.27 (47%)	3.82 (36%)	1.31 (86%)	Nil
73	M	54	1.86 (49%)	2.2 (47%)	3.44 (47%)	3.36 (31%)	1.18 (81%)	30, Stopped in 2006
37	M	62	1.58 (48%)	2.13 (37%)	4.43 (63%)	2.31 (24%)	0.63 (46%)	Nil
88	M	49	1.83 (51%)	2.87 (65%)	5.50 (81%)	4.6 (46%)	1.1 (73%)	Nil
90	M	62	2.07 (64%)	2.49 (61%)	3.54 (52%)	3.59 (39%)	1.10 (81%)	Nil
91	M	48	1.74 (50%)	2.08 (49%)	3.04 (46%)	2.26 (23%)	1.03 (69%)	10

Percentages of predicted values are in parentheses.

M, male; FEV1, forced expiratory volume in 1 second; FVC, forced vital capacity; IPF, idiopathic pulmonary fibrosis; KCO, carbon monoxide transfer coefficient; TLC, total lung capacity; TLCO, carbon monoxide transfer factor.

Taken together, these data show that the use of heterogeneous mixture of cells (ie, whole tissue) can mask the identity and expression patterns of miRNAs emanating from a particular, highly disease-relevant subpopulation of cells within the organ.

**Figure 4:**
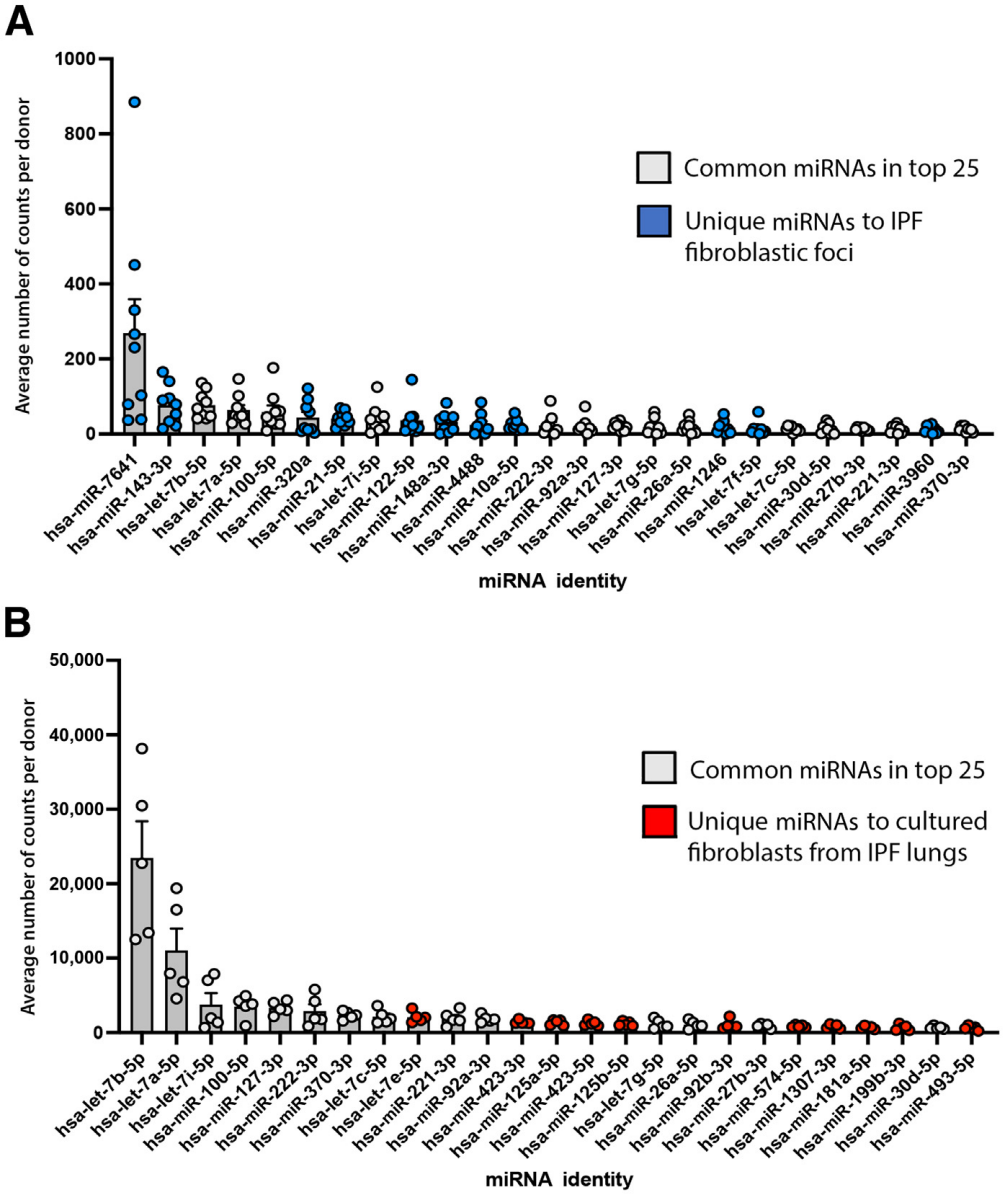
Most expressed miRNAs in fibroblastic foci and cultured primary fibroblasts isolated from idiopathic pulmonary fibrosis (IPF) lungs (counts). **A:** Top 25 most abundant miRNAs in IPF fibroblastic foci. miRNAs uniquely found in fibroblastic foci are indicated in **blue**. Common miRNAs found in fibroblastic foci and primary fibroblasts isolated from IPF lungs are indicated in **gray**. **B:** Top 25 most abundant miRNAs in cultured primary fibroblasts isolated from IPF lungs. miRNAs uniquely found in cultured fibroblasts isolated from IPF lungs are indicated in **red**.

**Figure 5:**
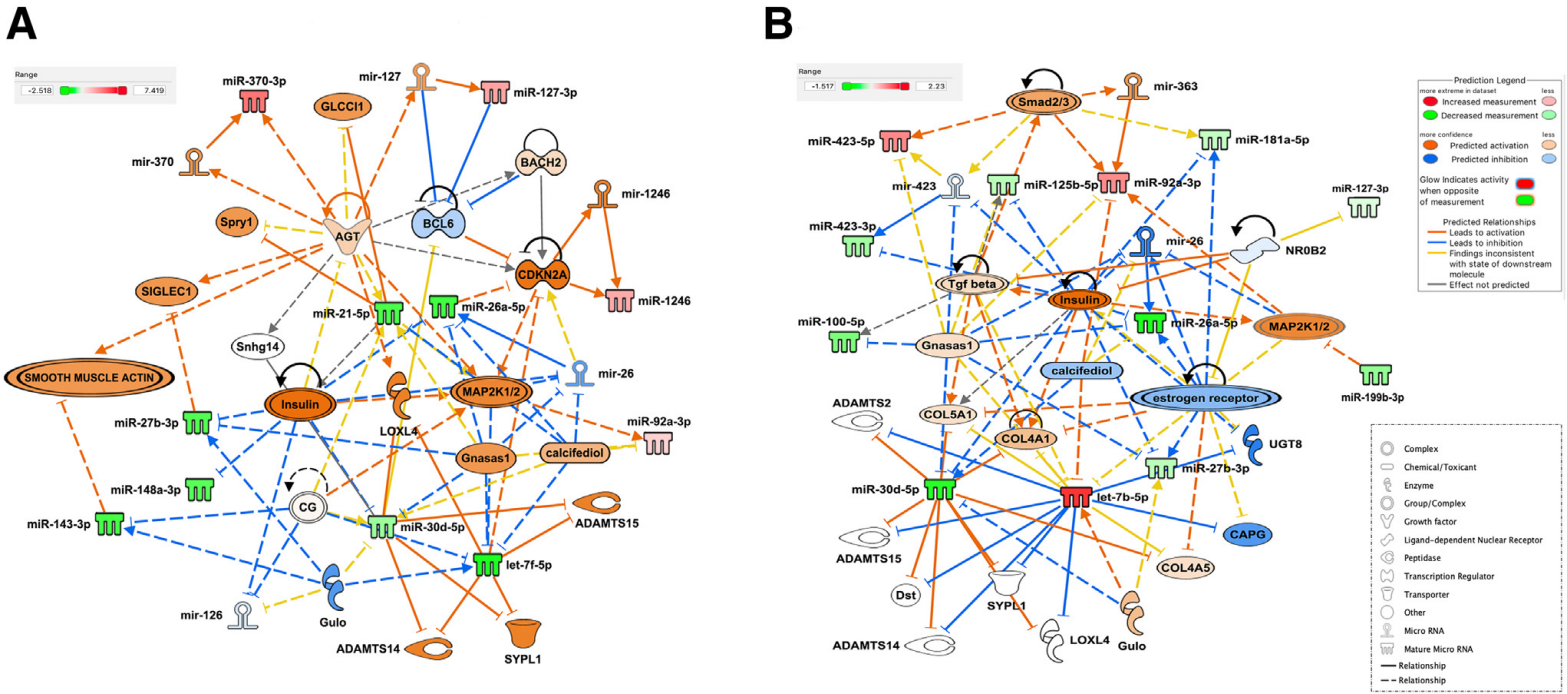
Most expressed miRNAs in fibroblastic foci and cultured primary fibroblasts isolated from idiopathic pulmonary fibrosis (IPF) lungs [Ingenuity Pathway Analysis (IPA) plots]. **A** and **B:** IPA-generated biological network of miRNAs with highest expression in fibroblastic foci and cultured primary IPF fibroblasts. Underexpressed and overexpressed miRNAs are indicated in **green** and **red**, respectively. Prediction of activation and inhibition is indicated in **orange** and **blue**, respectively. Molecules related to miRNAs for which no prediction of directional change could be made are indicated in **white**. **Lines** represent different relationships between the molecules and miRNAs, as stated in the legend.

**Supplemental Figure S1:**
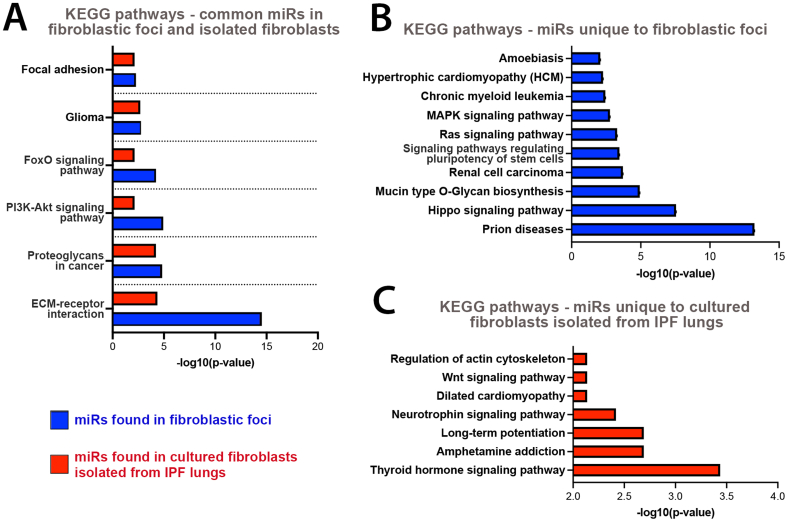
Enrichment Kyoto Encyclopedia of Genes and Genomes (KEGG) analysis of the top 25 most expressed miRNAs in fibrotic foci and cultured fibroblasts isolated from idiopathic pulmonary fibrosis (IPF) lung. **A:** KEGG pathways associated with 14 common miRNAs of the top 25 most expressed found in fibrotic foci (blue) and in cultured fibroblasts isolated from IPF lungs (red). The *x* axis shows the –log10 enrichment *P* value, whereas the *y* axis shows the different KEGG pathways. **B:** KEGG pathways associated with the unique 11 miRNAs of the top 25 found in fibrotic foci. The *x* axis shows the –log10 enrichment *P* value, whereas the *y* axis shows the different KEGG pathways. **C:** KEGG pathways associated with unique 11 miRNAs found in isolated fibroblasts of the top 25. The *x* axis shows the –log10 enrichment *P* value, whereas the *y* axis shows the different KEGG pathways. ECM, extracellular matrix; FoxO, forkhead box O1; MAPK, mitogen-activated protein kinase; PI3K, phosphatidylinositol 3-kinase.

Whether it was possible to determine the miRNA expression within FF by obtaining the miRNA profile of *in vitro* grown fibroblasts isolated from IPF lungs, given that FF predominantly contain fibroblasts/myofibroblasts, was studied next. To this end, fibroblasts were isolated from the same IPF donor lungs used for isolation of FF by LCM, and the miRNA profiles of *in vitro* grown cells were compared with LCM generated FF miRNAs. Of the 25 most abundant miRNA species in the two groups, this comparison identified 11 miRNAs exclusively in FF and 11 unique miRNAs in cultured fibroblasts (Figure 4). These data were used to identify the Kyoto Encyclopedia of Genes and Genomes pathways regulated by the miRNAs (Supplemental Figure S1). Although a significant number of Kyoto Encyclopedia of Genes and Genomes pathways affected by miRNAs were found in both FF and culture-grown fibroblasts (Supplemental Figure S1A), 10 pathways regulated by miRNAs were unique to FF (Supplemental Figure S1B) and an additional 7 Kyoto Encyclopedia of Genes and Genomes pathways mapped to miRNAs were identified solely in IPF fibroblasts grown *in vitro* (Supplemental Figure S1C). Ingenuity Pathway Analysis of the 25 most differentially expressed miRNAs indicated putative functions in several signaling pathways unique to FF (Figure 5). In summary, caution should be taken to interpret the data related to the biology of cultured IPF fibroblasts with respect to events occurring in the FF tissue microenvironment because of the dynamic loss of cell heterogeneity and plasticity.

**Figure 6:**
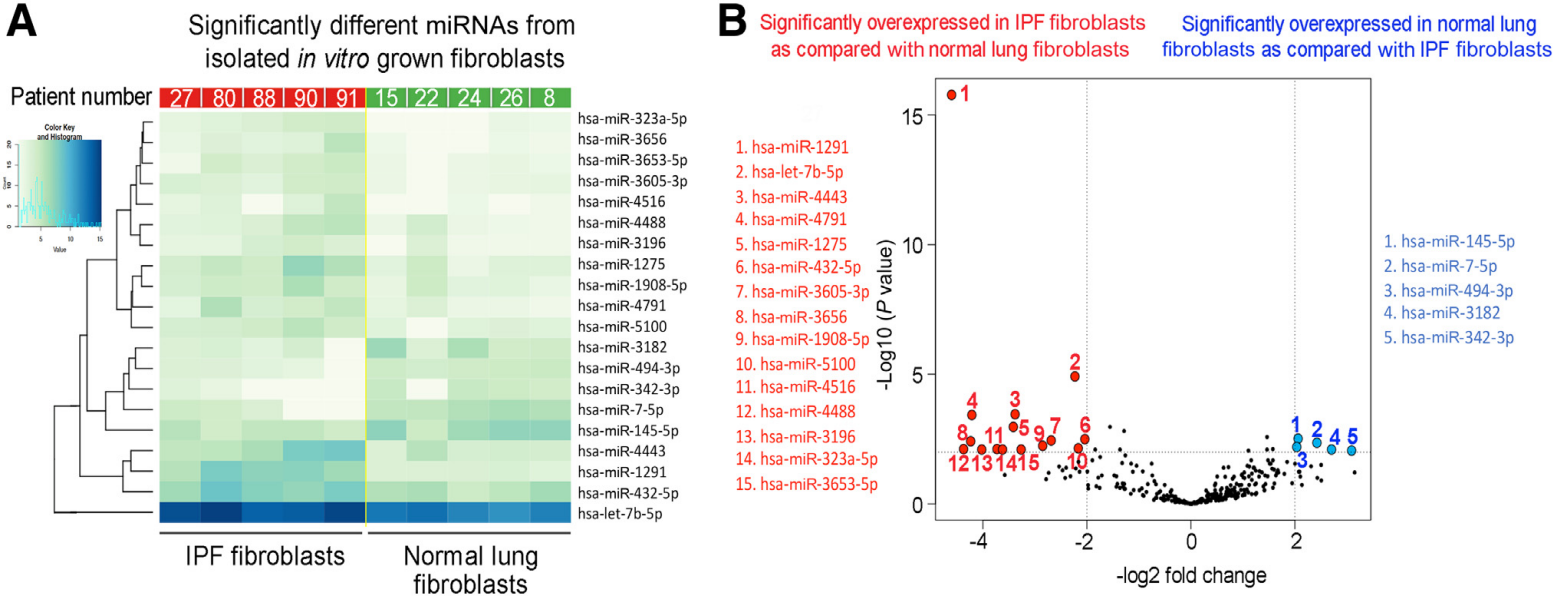
Differential expression of miRNAs in fibroblasts isolated from normal and idiopathic pulmonary fibrosis (IPF) lungs (heat map). **A:** Heat map of the 20 significant differentially expressed miRNAs in the comparison between IPF lung (**red;left side**) and normal lung primary cultured fibroblasts (**green;right side**); *P* < 0.01 and fold change >2. **B:** Volcano plot of the significant differentially expressed miRNAs comparing IPF lung and normal lung primary cultured fibroblasts. Five significantly overexpressed miRNAs in normal lung fibroblasts are indicated in **blue**. Fifteen significantly overexpressed miRNAs in IPF fibroblasts are indicated in **red**.

**Figure 7:**
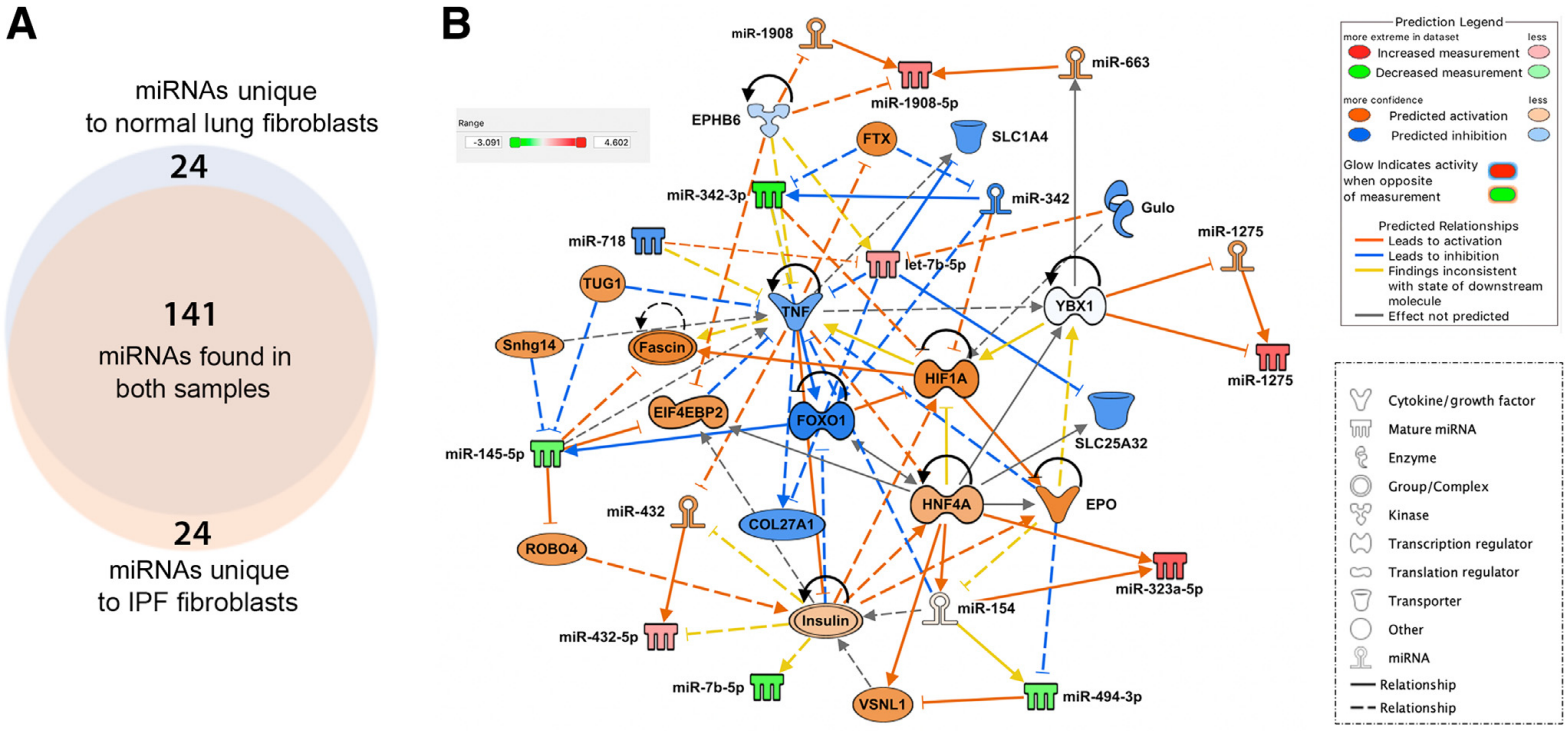
Differential expression of miRNAs in fibroblasts isolated from normal and idiopathic pulmonary fibrosis (IPF) lungs [Ingenuity Pathway Analysis (IPA) plot]. **A:** Venn diagram of the miRNAs with >10 counts in at least three samples per group. **Pale orange** indicates unique miRNAs in IPF fibroblasts, **pale blue** indicates unique miRNAs in fibroblasts from normal lungs, and **overlapping area** indicates miRNAs found in common in both fibroblasts isolated from normal and IPF lungs. **B:** IPA-generated biological network of differentially expressed miRNAs in fibroblasts isolated from normal and IPF lungs. Underexpressed and overexpressed miRNAs are indicated in **green** and **red**, respectively. Prediction of activation and inhibition is indicated in **orange** and **blue**, respectively. Molecules related to miRNAs for which no prediction of directional change could be made are indicated in **white**. **Lines** represent different relationships between the molecules and miRNAs, as stated in the legend.

**Figure 8:**
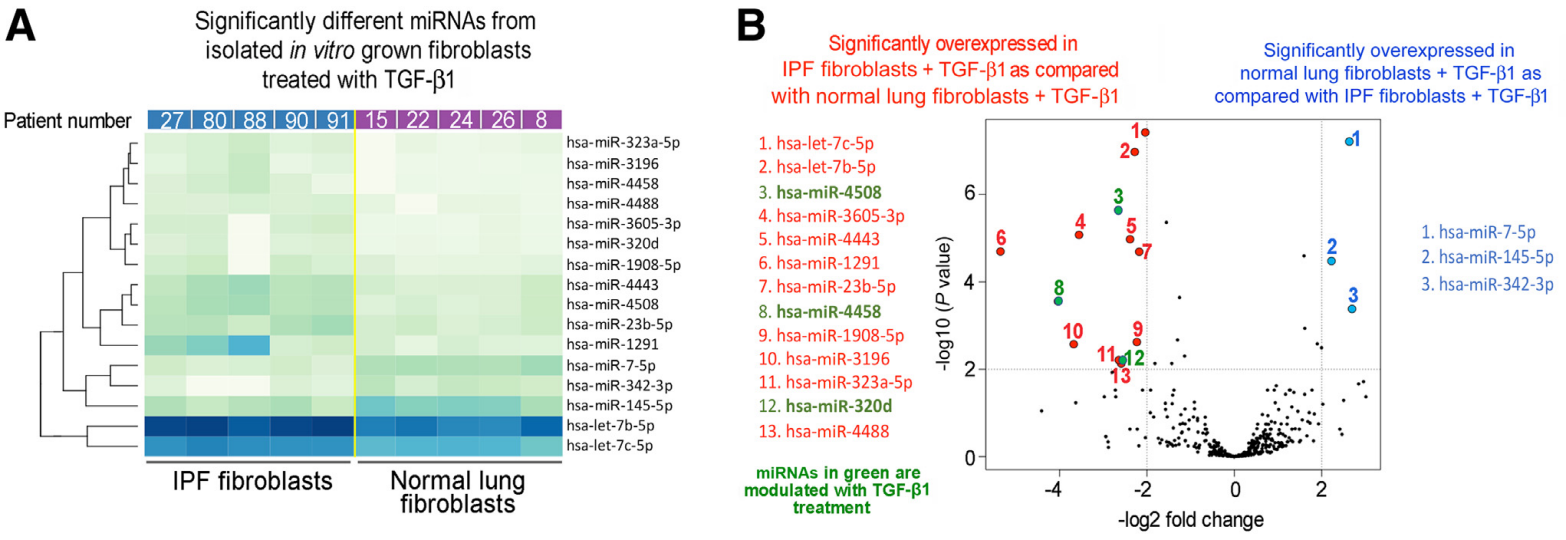
Differential expression of miRNAs in fibroblasts isolated from normal and idiopathic pulmonary fibrosis (IPF) lungs treated with transforming growth factor (TGF)-β1 (heat map). **A:** Heat map of the 16 significant differentially expressed miRNAs in the comparison between IPF lung (**blue;left side**) and normal lung primary cultured fibroblasts (**purple;right side**), both treated with TGF-β1; *P* < 0.01 and fold change >2. **B:** Volcano plot of the significant differentially expressed miRNAs comparing IPF lung and normal lung primary cultured fibroblasts treated with TGF-β1. Three significantly overexpressed miRNAs in normal lung fibroblasts treated with TGF-β1 are indicated in **blue**. Thirteen significantly overexpressed miRNAs in IPF fibroblasts treated with TGF-β1 are indicated in **red**. Three miRNAs that change as a result of treatment with TGF-β1 are indicated in **green**.

**Figure 9:**
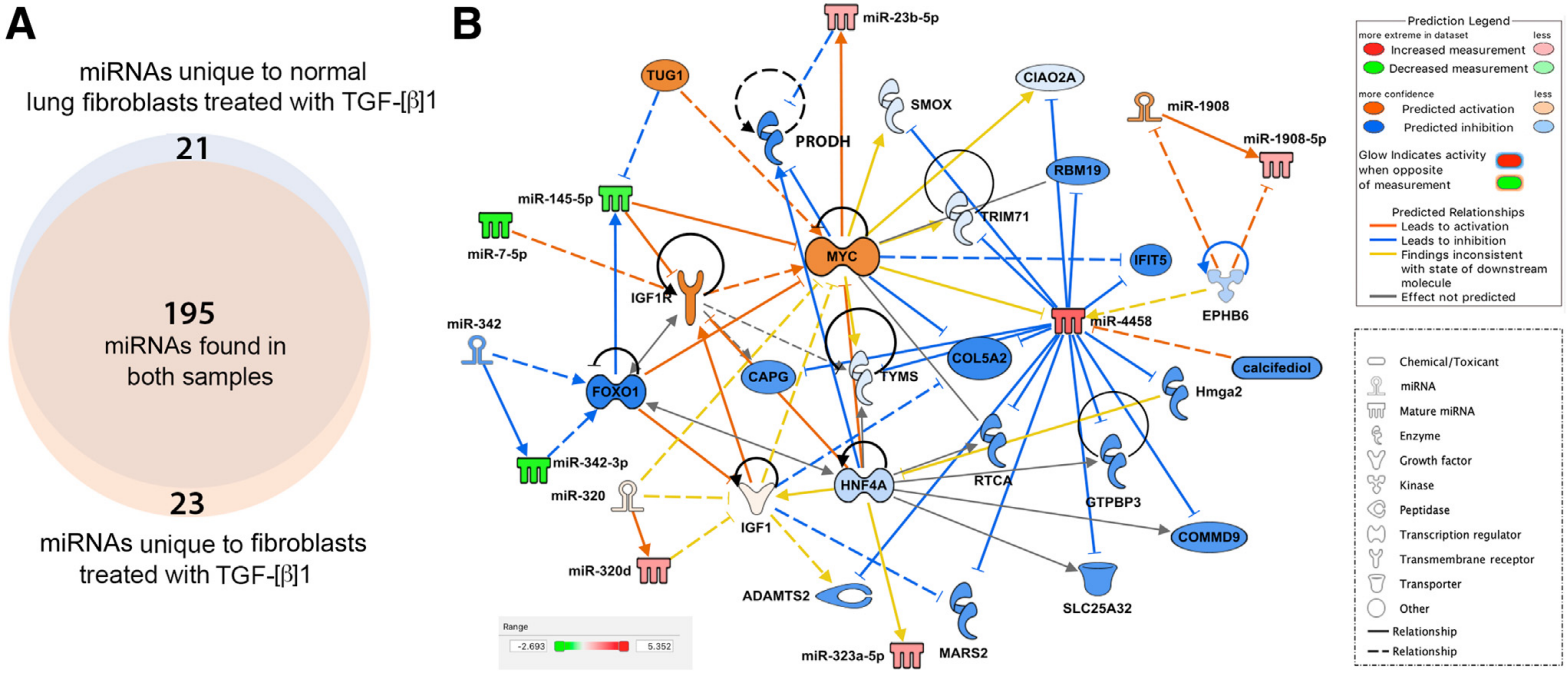
Differential expression of miRNAs in fibroblasts isolated from normal and idiopathic pulmonary fibrosis (IPF) lungs treated with transforming growth factor (TGF)-β1 (heat map). **A:** Venn diagram of the miRNAs with >10 counts in at least three samples per group. **Pale orange** indicates unique miRNAs in IPF fibroblasts treated with TGF-β1, **pale blue**, unique miRNAs in TGF-β–treated fibroblasts from normal lungs, and **overlapping area** indicates miRNAs found in common in both. **B:** Ingenuity Pathway Analysis–generated biological network of differentially expressed miRNAs in fibroblasts isolated from normal and IPF lungs, both treated with TGF-β1. Underexpressed and overexpressed miRNAs are indicated in **green** and **red**, respectively. Prediction of activation and inhibition is indicated in **orange** and **blue**, respectively. Molecules related to miRNAs for which no prediction of directional change could be made are indicated in **white**. **Lines** represent different relationships between the molecules and miRNAs, as stated in the legend.

Despite gene regulation alterations mediated by *in vitro* culture, isolated lung fibroblasts are a valuable resource in research. To further understand the limitations of *in vitro* culturing, whether fibroblasts cultured on plastic retain miRNA expression patterns that reflect the microenvironment and macroenvironment of the organ they were isolated from was studied next. To this end, fibroblasts from IPF lungs and/or normal lungs were isolated and cultured, and the expression profiles of miRNAs within the *in vitro* grown cells were compared (Figure 6A). Five miRNAs were significantly overexpressed in the normal lung fibroblasts (Supplemental Table S2 and Figure 6B) and 15 miRNAs were significantly overexpressed in IPF fibroblasts (Supplemental Table S2 and Figure 6B). These data also show a small difference in the fold change and *P* value between the IPF and normal lung fibroblasts, suggesting that the process of *in vitro* culturing may remodel the epigenome such that original tissue of origin signatures is at least diminished, if not completely lost. Moreover, when considering miRNAs with >10 counts in at least three samples per group, 141 miRNAs were found to be overlapping, whereas 24 miRNAs were present only in normal cells and 24 were unique to IPF cells (Figure 7A). Ingenuity Pathway Analysis revealed that significant differentially expressed miRNAs in fibroblasts from IPF lungs were found to be related to the transcription regulators [hypoxia inducible factor 1 subunit alpha (HIF1A), hepatocyte nuclear factor 4 alpha (HNF4A), Y-box binding protein 1 (YBX1), and forkhead box O1 (FOXO1)], the translation regulator eukaryotic translation initiation factor 4E binding protein 2 (EIF4EBP2), other miRNAs (miR-1275, miR-663, miR-1908, miR-342, miR-432, and miR-154), solute carrier family 1 member 4 (SLC1A4) and SLC25A32 transporters, Gulo enzyme, cytokine (tumor necrosis factor and erythropoietin), EPHB6 kinase, insulin, roundabout guidance receptor 4 (ROBO4), and other features [taurine up-regulated 1 (TUG1); Fascin; Snhg14; visinin like 1 (VSNL1); FTX transcript, XIST regulator (FTX); and collagen type 27 alpha 1 (COL27A1)] (Figure 7B). To further explore the biological behavior of IPF and normal lung fibroblasts, the cells were treated with TGF-β1 to ascertain the response to profibrogenic stimulus; miRNAs were then isolated and sequenced (Figure 8A). The comparison identified 13 miRNAs that were significantly overexpressed in the IPF fibroblasts treated with TGF-β1 (Supplemental Table S3 and Figure 8B) and 3 miRNAs that were significantly repressed compared with the fibroblasts from normal lungs treated with TGF-β1 (Supplemental Table S3 and Figure 8B). Closer inspection revealed that 3 of 13 significantly overexpressed miRNAs in the IPF lung fibroblasts are due to treatment with TGF-β1 (Figure 8B), with the other 10 miRNAs present as baseline difference between the IPF and normal lung fibroblasts (Figure 8B). Likewise, the remaining three overexpressed miRNAs in normal lung fibroblasts were present at baseline (Figure 8B). Of the 239 TGF-β1–dependent differentially regulated miRNAs in cultured lung fibroblasts, 195 were common to normal and IPF fibroblasts, whereas 21 were unique to normal fibroblasts and 23 were unique to the IPF cultured fibroblasts (Figure 9A). Ingenuity Pathway Analysis showed these significant miRNAs to be associated with several features, some of which were also found related to miRNAs differently expressed in fibroblasts from IPF lungs, such the transporter SLC25A32, TUG1, EPHB6 kinase, miRNAs (miR-342 and miR-1908), and the transcription regulators FOXO1 and HNF4A. In addition, the relationship with different molecules was also revealed, including miR-320, the peptidase ADAMTS2, insulin like growth factor 1 (IGF1), several enzymes [methionyl-TRNA synthetase 2, mitochondrial (MARS2); thymidylate synthetase (TYMS); tripartite motif containing 71 (TRIM71); spermine oxidase (SMOX); high mobility group AT-hook 2 (Hmga2); GTP binding protein 3, mitochondrial (GTPBP3); RNA 3′-terminal phosphate cyclase (RTCA); and proline dehydrogenase 1 (PRODH)], the transcription regulator MYC proto-oncogene, BHLH transcription factor (MYC), calcifediol, and other molecules [interferon-induced protein with tetratricopeptide repeats 5 (IFIT5); RNA binding motif protein 19 (RBM19); COMM domain containing 9 (COMMD9); cytosolic iron-sulfur assembly component 2A (CIAO2A); COL5A2; and capping actin protein, gelsolin like (CAPG)] (Figure 9B). These data further confirm that the *in vitro* culture of fibroblasts is a potent epigenetic remodeler such that even the treatment with TGF-β1, a potent inducer of fibrogenic signaling, has little effect. These data show that culturing on plastic may alter miRNA expression levels in primary fibroblasts such that organ of origin epigenetic signatures may be diminished or erased.

## Discussion

miRNAs are short noncoding RNA molecules that function in post-transcriptional regulation of gene expression. Along with other epigenetic mechanisms, miRNAs regulate gene expression in physiological and pathophysiological conditions. This study addressed the role of miRNAs in the pathophysiology of IPF by investigating their expression patterns within fibroblastic foci, the hallmark IPF lesions highly rich in extracellular matrix–producing cell types, such as fibroblasts and myofibroblasts. Extracellular matrix–generating activities of these cells are of cardinal importance in development of IPF; therefore, understanding the underlying epigenetic regulation may illuminate novel ways of manipulating disease progression.

To this end, fibroblastic foci were isolated from IPF lungs using laser microdissection. This approach generated a pure population of cells that were fixed *in situ*, thus preserving all of the miRNA epigenetic signatures determined by microenvironmental influences, including those linked to the disease process itself. From these signatures, it was possible to gain insight into novel signaling pathways and genes affected by miRNAs identified. More importantly, the current data show that it is not possible to gain equivalent understanding from either the isolated fibroblasts or whole IPF lung tissue. As such, these findings represent significant and useful incremental gain in our knowledge of the subject.

Previous studies collectively identified several miRNAs commonly found in IPF tissue or IPF fibroblasts, with these being the members of let-7 family (let-7a, let-7b, let-7c, let-7d, let-7e, let-7f, let-7g, let-7I, and miR-98), miR-21, miR-9, miR-26a, miR-29a, miR-30 family, miR-145, miR-155, miR-200a-c, miR-375, miR-326, miR-153, and miR-1343. Herein, the presence of most of these miRNAs was confirmed and additional species present in IPF lungs, fibroblastic foci, and IPF cultured fibroblasts were uncovered.

It is well established that epigenetic determinants, including miRNAs, are exquisitely cell type–specific. Previously published studies used either whole IPF lung tissue or isolated and cultured lung fibroblasts to determine miRNA expression. Fibroblastic foci within the IPF lung almost exclusively contain fibroblasts/myofibroblasts, which are the cell types known to drive IPF progression. Therefore, whether a heterogeneous mixture of lung cells (as found in a whole lung) can be used to learn about processes that regulate progression of IPF, given that those processes are thought to be driven by events emanating from the FF, was investigated.

This study addressed several outstanding important questions:

i) Can novel and valuable insight be gained by determining epigenetic signatures that are unique to FF in IPF lungs?
ii) FF almost exclusively contain fibroblasts/myofibroblasts; therefore, is it possible to understand the miRNA expression within FF by isolating fibroblasts, growing them *in vitro*, and assessing their miRNA content?
iii) Do fibroblasts cultured on plastic retain miRNA expression patterns that reflect the microenvironment and macroenvironment of the organ they were isolated from, or does culturing lung fibroblasts on plastic cause major alterations in miRNA expression?

Interestingly, only five common miRNAs were found to be significantly different in FF and IPF fibroblasts: miR-145-5p, miR-4443, miR-4488, miR-4516, and miR-5100. Of these, miR-145-5p and miR-5100 have previously been described in IPF.^12^^,^^19^, ^20^, ^21^ However, to date, miR-4443, miR-4488, and miR-4516 have not been shown to be related to IPF.

Previous studies identified miR-4443 as a key regulator of tumorigenesis and metastasis in a variety of tumors.^22^, ^23^, ^24^, ^25^ In particular, miR-4443 up-regulation in lung has been related to chemotherapy resistance in patients with non–small-cell lung cancer.^26^^,^^27^ The results of Zhang et al^27^ suggested that the overexpression of miR-4443 promoted the resistance to epirubicin-based chemotherapy of non–small-cell lung cancer cells via the activation of the Janus kinase 2 (JAK2)/STAT3 pathway. Moreover, Chowdhari and Saini^28^ identified miR-4516 as negative regulator of STAT3 in cultured human keratinocytes. Interestingly, the JAK2/STAT3 pathway has been associated with fibrosis, including pulmonary fibrosis.^29^, ^30^, ^31^ The current results show miR-4443 and miR-4516 to be up-regulated in both FF and IPF fibroblasts.

Up-regulation of miR-4488 was observed in the exosome of patients with dermatomyositis complicated with interstitial lung diseases before treatment, when compared with patients with dermatomyositis without prior interstitial lung disease complications. Bioinformatic analysis of the putative miR-4488 targets suggested that miR-4488 may contribute to systemic inflammation in patients with dermatomyositis complicated with interstitial lung diseases.^32^ However, miR-4488 implication in IPF has not been described.

Interestingly, 38 of 43 miRNAs were found to be expressed only in FF, but not in IPF, fibroblasts, either with or without TGF-β1 stimulation. From these 38 miRNAs, 25 have been already described in IPF (Table 2^12^^,^^19^, ^20^, ^21^^,^^33^, ^34^, ^35^, ^36^, ^37^, ^38^, ^39^, ^40^, ^41^, ^42^, ^43^, ^44^, ^45^, ^46^, ^47^, ^48^, ^49^, ^50^, ^51^, ^52^, ^53^, ^54^, ^55^, ^56^, ^57^, ^58^, ^59^); however, to the best of our knowledge, 13 have never been described as related to IPF. Among these, several miRNAs have been described in fibrotic disease in other tissues. For instance, miR-370-3p, miR-222-3p, miR-146a-5p, and miR-203a-3p, among others (Table 2), have been described in liver fibrosis; and miR-4454 and miR-23a-3p have been linked to cardiac and renal fibrosis (Table 2). Of note, miR-4448 and miR-4284 have no obvious links to IPF or fibrosis, and given their expression in FF, it will therefore be instructive to examine these miRNAs for potential novel functions in IPF. Altogether, these findings suggest that the use of whole organ or *in vitro* grown fibroblast likely masks microenvironmental epigenetic changes occurring within the FF.

**Table 2:** Fibroblastic Foci from IPF Lungs Compared with Whole IPF Lung Tissue

Variable	miRNAs	Fibrosis	Reference
Up-regulated in FF	hsa-miR-122-5p	IPF	33
	hsa-miR-127-3p	IPF	34
	hsa-miR-370-3p	HSC	35
	hsa-miR-423-5p	IPF	34,36
	hsa-miR-222-3p	HSC	35,37
	hsa-miR-4488		
	hsa-miR-320a	IPF	38
	hsa-miR-7641	HSC	35
	hsa-miR-1246	HSC	35
	hsa-let-7e-5p	HSC	35
	hsa-miR-221-3p	IPF	39
	hsa-miR-4508	HSC and cirrhosis	35,40
	hsa-miR-4516		
	hsa-miR-4497	HSC	35
	hsa-miR-192-5p	IPF	41
	hsa-miR-4443		
	hsa-miR-203a-3p	HSC	35,42
	hsa-miR-4448		
Down-regulated in FF	hsa-miR-99a-5p	IPF	19,34
	hsa-miR-126-3p	IPF	34
	hsa-miR-21-5p	IPF	12,19,35,41,43
	hsa-let-7f-5p	IPF	44
	hsa-miR-27a-3p	IPF	12,19,45
	hsa-miR-34c-5p	IPF	19,34,46
	hsa-miR-30a-5p	IPF	19,34,47,48
	hsa-miR-143-3p	IPF	19
	hsa-miR-4284		
	hsa-miR-4454	Cardiac fibrosis	49
	hsa-miR-26a-5p	IPF	12,19,34,50
	hsa-miR-26b-5p	IPF	19,34
	hsa-miR-451a	IPF	34
	hsa-miR-145-5p	IPF	12,19,20
	hsa-miR-5100	IPF	21
	hsa-miR-146a-5p	HSC	35,51
	hsa-miR-24-3p	IPF	52
	hsa-miR-30b-5p	IPF	34
	hsa-miR-101-3p	IPF	23,53
	hsa-miR-143-5p	IPF	19
	hsa-miR-200c-3p	IPF	19,54,55
	hsa-miR-200b-3p	IPF	19,54,55
	hsa-miR-23a-3p	HSC and renal fibrosis	35,56
	hsa-miR-30a-3p	IPF	57,58
	hsa-miR-199a-5p	IPF	34,59

List includes miRNAs that are significant in FF, primary fibroblasts, and/or primary fibroblasts + transforming growth factor-β1.

FF, fibroblastic foci; HSC, hepatic stellate cell; IPF, idiopathic pulmonary fibrosis.

In summary, by analyzing miRNA expression specifically in FF, the study was able to identify up to 38 miRNAs that were exclusively expressed in FF, which could potentially contribute to IPF development and progression. Future studies into biology of IPF should utilize platforms that better reflect the distinct biology of the FF tissue niche, such as precision cut IPF lung slices that may be a more suitable preclinical model that closely recapitulates *in vivo* environment of diseased lung.
