# Supplementary material for: miRNA Expression in Fibroblastic Foci within Idiopathic Pulmonary Fibrosis Lungs Reveals Novel Disease-Relevant Pathways
Source: Am J Pathol. 2023 Jan 20;193(4):417–29. doi: 10.1016/j.ajpath.2022.12.015 (PMC12178335; doi:10.1016/j.ajpath.2022.12.015)
Supplement: Supplemental Table S1 [file mmc1.docx]

**Supplementary Table 1 – IPF fibroblastic foci vs IPF whole lung tissue**

**miRs overexpressed in whole IPF lung as compared to fibroblastic foci**

| **miRNAs** | **log2FoldChange** | **lfcSE** | **pvalue** | **padj** |
| --- | --- | --- | --- | --- |
| hsa-mir-34c-5p | -6.055659 | 0.91239867 | 3.20 X 10^-11^ | 7.12 X 10^-10^ |
| hsa-mir-126-3p | -4.698401 | 0.58057396 | 5.84 X 10^-16^ | 3.46 X 10^-14^ |
| hsa-mir-146a-5p | -4.6541354 | 1.03667984 | 7.14 X 10^-6^ | 4.85 X 10^-5^ |
| hsa-mir-4454 | -4.5547527 | 0.83373895 | 4.68 X 10^-8^ | 5.95 X 10^-7^ |
| hsa-mir-451a | -4.4491005 | 0.87697491 | 3.91 X 10^-7^ | 3.36 X 10^-6^ |
| hsa-mir-4284 | -4.087511 | 0.73729555 | 2.96 X 10^-8^ | 4.05 X 10^-7^ |
| hsa-mir-27a-3p | -3.9480965 | 0.55739581 | 1.41 X 10^-12^ | 4.18 X 10^-11^ |
| hsa-mir-30b-5p | -3.4667383 | 0.86849526 | 6.56 X 10^-5^ | 0.0003435 |
| hsa-mir-145-5p | -3.4238917 | 0.74788296 | 4.69 X 10^-6^ | 3.48 X 10^-5^ |
| hsa-mir-99a-5p | -3.2898098 | 0.398049 | 1.40 X 10^-16^ | 1.24 X 10^-14^ |
| hsa-mir-200c-3p | -3.275709 | 0.96737709 | 0.00070874 | 0.0030037 |
| hsa-mir-143-5p | -3.2406482 | 0.87799017 | 0.00022339 | 0.00104639 |
| hsa-mir-26b-5p | -3.0263048 | 0.57301495 | 1.28 X 10^-7^ | 1.34 X 10^-6^ |
| hsa-mir-101-3p | -2.9763814 | 0.76449482 | 9.89 X 10^-5^ | 0.00048902 |
| hsa-mir-200b-3p | -2.814614 | 0.8644367 | 0.0011299 | 0.00457096 |
| hsa-mir-5100 | -2.5947076 | 0.57875538 | 7.35 X 10^-6^ | 4.85 X 10^-5^ |
| hsa-let-7f-5p | -2.5182563 | 0.35080747 | 7.05 X 10^-13^ | 2.51 X 10^-11^ |
| hsa-mir-30a-5p | -2.5125537 | 0.3816144 | 4.58 X 10^-11^ | 9.06 X 10^-10^ |
| hsa-mir-23a-3p | -2.4541879 | 0.78830827 | 0.00185049 | 0.00716061 |
| hsa-mir-30a-3p | -2.2156748 | 0.72642377 | 0.00228755 | 0.008483 |
| hsa-mir-24-3p | -2.1745336 | 0.48649194 | 7.83 X 10^-6^ | 4.98 X 10^-5^ |
| hsa-mir-199a-5p | -2.1473504 | 0.71613288 | 0.0027128 | 0.00985466 |
| hsa-mir-26a-5p | -2.1241956 | 0.39921694 | 1.03 X 10^-7^ | 1.15 X 10^-6^ |
| hsa-mir-143-3p | -2.0576813 | 0.36852236 | 2.36 X 10^-8^ | 3.49 X 10^-7^ |
| hsa-mir-21-5p | -2.0044029 | 0.26603512 | 4.91 X 10^-14^ | 2.18 X 10^-12^ |

**miRs overexpressed in fibroblastic foci as compared to whole IPF lung**

| **miRNAs** | **log2FoldChange** | **lfcSE** | **pvalue** | **padj** |
| --- | --- | --- | --- | --- |
| hsa-mir-122-5p | 7.41932199 | 0.82158961 | 1.71 X 10^-19^ | 3.04 X 10^-17^ |
| hsa-mir-370-3p | 4.95274527 | 0.8193348 | 1.50 X 10^-9^ | 2.46 X 10^-8^ |
| hsa-mir-4488 | 4.12736128 | 0.81302484 | 3.84 X 10^-7^ | 3.36 X 10^-6^ |
| hsa-mir-222-3p | 3.50979849 | 0.69219313 | 3.97 X 10^-7^ | 3.36 X 10^-6^ |
| hsa-mir-4516 | 3.35847176 | 0.85095942 | 7.92 X 10^-5^ | 0.00040299 |
| hsa-mir-127-3p | 3.29868672 | 0.54594706 | 1.52 X 10^-9^ | 2.46 X 10^-8^ |
| hsa-mir-192-5p | 3.27683173 | 0.93936682 | 0.00048603 | 0.00221828 |
| hsa-mir-423-5p | 3.19208114 | 0.62404267 | 3.13 X 10^-7^ | 3.10 X 10^-6^ |
| hsa-mir-4448 | 3.12091673 | 1.02296782 | 0.00228198 | 0.008483 |
| hsa-mir-1246 | 3.08763978 | 0.68034063 | 5.67 X 10^-6^ | 4.04 X 10^-5^ |
| hsa-mir-4508 | 3.05751691 | 0.75872528 | 5.58 X 10^-5^ | 0.0003011 |
| hsa-mir-320a | 3.00436871 | 0.59731607 | 4.91 X 10^-7^ | 3.97 X 10^-6^ |
| hsa-mir-203a-3p | 2.96749436 | 0.92957393 | 0.00141136 | 0.00558273 |
| hsa-mir-4497 | 2.62363886 | 0.69062958 | 0.00014533 | 0.00069918 |
| hsa-mir-221-3p | 2.52611044 | 0.61278953 | 3.75 X 10^-5^ | 0.00020864 |
| hsa-let-7 x 105p | 2.34623847 | 0.53390574 | 1.11 X 10^-5^ | 6.59 X 10^-5^ |
| hsa-mir-7641 | 2.31286513 | 0.49555931 | 3.05 X 10^-6^ | 2.36 X 10^-5^ |
| hsa-mir-4443 | 2.30785173 | 0.67136238 | 0.00058697 | 0.00254831 |
