# Supplementary material for: miRNA Expression in Fibroblastic Foci within Idiopathic Pulmonary Fibrosis Lungs Reveals Novel Disease-Relevant Pathways
Source: Am J Pathol. 2023 Jan 20;193(4):417–29. doi: 10.1016/j.ajpath.2022.12.015 (PMC12178335; doi:10.1016/j.ajpath.2022.12.015)
Supplement: Supplemental Table S2 [file mmc2.docx]

**Supplementary Table 2 – IPF fibroblasts vs normal lung fibroblasts**

**miRNAs overexpressed in IPF fibroblasts as compared to normal lung fibroblasts**

| **miRNAs** | **log2FoldChange** | **lfcSE** | **pvalue** | **padj** |
| --- | --- | --- | --- | --- |
| hsa-mir-1291 | 4.60198455 | 0.51738406 | 5.86 X 10^-19^ | 1.68 X 10^-16^ |
| hsa-let-7b-5p | 2.22982354 | 0.41627438 | 8.48 X 10^-8^ | 1.22 X 10^-5^ |
| hsa-mir-4516 | 4.37177722 | 1.25003631 | 0.00046996 | 0.00770685 |
| hsa-mir-3656 | 4.23454069 | 1.12206517 | 0.00016073 | 0.00384406 |
| hsa-mir-4791 | 4.21237223 | 0.92431274 | 5.18 X 10^-6^ | 0.00037177 |
| hsa-mir-3196 | 4.02279492 | 1.18885429 | 0.00071501 | 0.00801945 |
| hsa-mir-4488 | 3.72886731 | 1.06976359 | 0.00049086 | 0.00770685 |
| hsa-mir-323a-5p | 3.62240527 | 1.05687944 | 0.00060927 | 0.00801945 |
| hsa-mir-1275 | 3.41504054 | 0.80559549 | 2.24 X 10^-5^ | 0.00107322 |
| hsa-mir-4443 | 3.38101997 | 0.73022308 | 3.65 X 10^-6^ | 0.00034962 |
| hsa-mir-3653-5p | 3.2647465 | 0.95973605 | 0.00066965 | 0.00801945 |
| hsa-mir-1908-5p | 2.85039824 | 0.78460885 | 0.00028026 | 0.00574541 |
| hsa-mir-3605-3p | 2.68678961 | 0.70461748 | 0.00013722 | 0.00358031 |
| hsa-mir-5100 | 2.16560413 | 0.61183635 | 0.00040086 | 0.00719047 |
| hsa-mir-432-5p | 2.03923857 | 0.5276333 | 0.00011114 | 0.00318985 |

**miRNAs overexpressed in normal lung fibroblasts as compared to IPF fibroblasts**

| **miRNAs** | **log2FoldChange** | **lfcSE** | **pvalue** | **padj** |
| --- | --- | --- | --- | --- |
| hsa-mir-342-3p | -3.0908209 | 0.92595777 | 0.00084392 | 0.00880128 |
| hsa-mir-3182 | -2.7046177 | 0.78628276 | 0.00058225 | 0.00801945 |
| hsa-mir-7-5p | -2.4236231 | 0.6514504 | 0.00019895 | 0.00439215 |
| hsa-mir-145-5p | -2.0615665 | 0.52846498 | 9.58 X 10^-5^ | 0.00305424 |
| hsa-mir-494-3p | -2.0363722 | 0.56755605 | 0.00033327 | 0.00637651 |
