# Supplementary material for: miRNA Expression in Fibroblastic Foci within Idiopathic Pulmonary Fibrosis Lungs Reveals Novel Disease-Relevant Pathways
Source: Am J Pathol. 2023 Jan 20;193(4):417–29. doi: 10.1016/j.ajpath.2022.12.015 (PMC12178335; doi:10.1016/j.ajpath.2022.12.015)
Supplement: Supplemental Table S3 [file mmc3.docx]

**Supplementary Table 3 – TGFβ1 treated IPF fibroblasts vs TGFβ1 treated normal lung fibroblasts**

**miRNAs overexpressed in IPF fibroblasts + TGFβ1 as compared to normal lung fibroblasts + TGFβ1**

| **miRNAs** | **log2FoldChange** | **lfcSE** | **pvalue** | **padj** |
| --- | --- | --- | --- | --- |
| hsa-mir-1291 | 5.35228609 | 1.05764784 | 4.18 X 10^-7^ | 2.03 X 10^-5^ |
| hsa-mir-4458 | 4.03608084 | 0.91056389 | 9.31 X 10^-6^ | 0.00027871 |
| hsa-mir-3196 | 3.6765126 | 0.96104688 | 0.00013049 | 0.00267164 |
| hsa-mir-3605-3p | 3.55454626 | 0.67344012 | 1.30 X 10^-7^ | 8.46 X 10^-6^ |
| hsa-mir-4508 | 2.65766261 | 0.47602764 | 2.36 X 10^-8^ | 2.30 X 10^-6^ |
| hsa-mir-323a-5p | 2.64426519 | 0.74144226 | 0.00036195 | 0.00616123 |
| hsa-mir-4488 | 2.58800781 | 0.73930536 | 0.00046422 | 0.00740185 |
| hsa-mir-320d | 2.5539364 | 0.71645429 | 0.00036429 | 0.00616123 |
| hsa-mir-4443 | 2.38651463 | 0.45831899 | 1.92 X 10^-7^ | 1.07 X 10^-5^ |
| hsa-let-7b-5p | 2.2793699 | 0.37121047 | 8.23 X 10^-10^ | 1.07 X 10^-7^ |
| hsa-mir-1908-5p | 2.23077486 | 0.57486742 | 0.00010424 | 0.00238527 |
| hsa-mir-23b-5p | 2.17703122 | 0.4323165 | 4.76 X 10^-7^ | 2.06 X 10^-5^ |
| hsa-let-7c-5p | 2.03777831 | 0.31503174 | 9.90 X 10^-11^ | 3.85 X 10^-8^ |

**miRNAs overexpressed in normal lung fibroblasts + TGFβ1 as compared to IPF fibroblasts + TGFβ1**

| **miRNAs** | **log2FoldChange** | **lfcSE** | **pvalue** | **padj** |
| --- | --- | --- | --- | --- |
| hsa-mir-342-3p | -2.6925829 | 0.62204096 | 1.50 X 10^-5^ | 0.0004169 |
| hsa-mir-7-5p | -2.630805 | 0.41829121 | 3.19 X 10^-10^ | 6.20 X 10^-8^ |
| hsa-mir-145-5p | -2.2223775 | 0.4533665 | 9.49 X 10^-7^ | 3.36 X 10^-5^ |
